# Lack of predictive tools for conventional and targeted cancer therapy: Barriers to biomarker development and clinical translation^☆^

**DOI:** 10.1016/j.addr.2021.113854

**Published:** 2021-09

**Authors:** Nikolaos Batis, Jill M. Brooks, Karl Payne, Neil Sharma, Paul Nankivell, Hisham Mehanna

**Affiliations:** a Institute of Head and Neck Studies and Education (InHANSE), Institute of Cancer and Genomic Sciences, College of Medical and Dental Sciences, University of Birmingham, Birmingham, United Kingdom; b Department of Head and Neck Surgery, Queen Elizabeth Hospital Birmingham, Birmingham, United Kingdom

**Keywords:** Predictive biomarker, Predictive tool, Predictive signature, Treatment response, Liquid biopsy, Multi-omics, Trial design, Intra-tumoral heterogeneity, Tumor microenvironment, AJCC, American Joint Committee on Cancer, BRC, breast cancer, CDx, companion diagnostic, CRC, colorectal cancer, CTC, circulating tumor cell, ctDNA, circulating tumor DNA, ERCC1, excision repair cross completing group 1, ESMO, European Society for Medical Oncology, FDA, Food and Drug Administration (US), FFPE, formalin-fixed, paraffin-embedded, HNSCC, head and neck squamous cell carcinoma, HPV, human papilloma virus, ITH, intra-tumoral heterogeneity, NGS, next generation sequencing, NSCLC, non-small cell lung cancer, PD-L1, programmed death ligand 1, PET-CT, positron emission tomography-computed tomography, TKI, tyrosine kinase inhibitor, TME, tumor microenvironment, TNM, tumor node metastasis

## Abstract

Predictive tools, utilising biomarkers, aim to objectively assess the potential response to a particular clinical intervention in order to direct treatment. Conventional cancer therapy remains poorly served by predictive biomarkers, despite being the mainstay of treatment for most patients. In contrast, targeted therapy benefits from a clearly defined protein target for potential biomarker assessment.

We discuss potential data sources of predictive biomarkers for conventional and targeted therapy, including patient clinical data and multi-omic biomarkers (genomic, transcriptomic and protein expression). Key examples, either clinically adopted or demonstrating promise for clinical translation, are highlighted. Following this, we provide an outline of potential barriers to predictive biomarker development; broadly discussing themes of approaches to translational research and study/trial design, and the impact of cellular and molecular tumor heterogeneity. Future avenues of research are also highlighted.

## Graphical abstract

**Figure:**
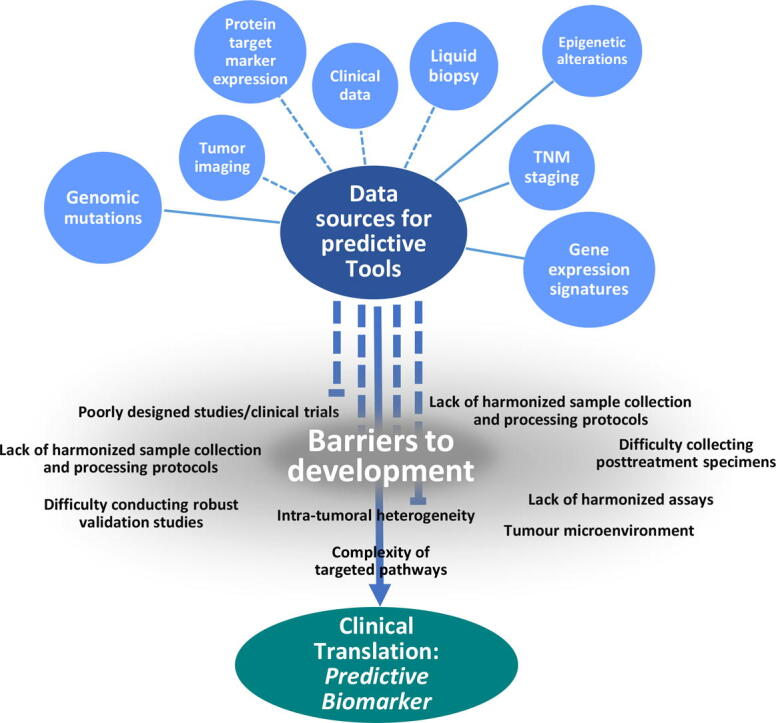

## 1 Introduction

Predictive tools aim to objectively assess the potential response to a particular clinical intervention or evaluate the differential outcomes – including toxicity – of two or more interventions, in order to direct treatment. Their use in guiding treatment decisions should therefore lead to improved clinical outcomes. In contrast, prognostic tools (or biomarkers) provide information about likely patient outcome irrespective of treatment [1]. Predictive tools for oncology can be derived from various data sources, including patient clinical details, histologic or radiologic data and multiple omic biomarkers (genomic, transcriptomic, epigenomic and protein expression). Hence, in this article we use the term predictive tools to include all the above, but will focus on predictive omic biomarkers, predictive analytics, and nomograms.

Clinicopathological data remains the primary stratification method used by clinicians when prescribing conventional anti-cancer therapy. Clinicopathological markers include tumor clinical and imaging-based assessment and histopathological findings, including tumor protein marker expression. Pathological stage, as defined by the AJCC TNM criteria (as a combination of *T*umor size, *N*odal and distant *M*etastasis) is the widely adopted method of prognostic risk stratification across solid tumors [2]. The most recent 8th Edition of the AJCC criteria demonstrated a significant shift in focus through the incorporation of molecular biomarkers into TNM staging for some cancer types. For example, expression of key receptors (HER2, oestrogen, progesterone) and genes (OncotypeDx score) are incorporated into a pathologic stage group for breast cancer [3] and expression of p16 protein – a surrogate marker of human papilloma virus infection – is included for oropharyngeal cancer [4]. However, whilst molecular subtyping may predict response to individual treatments, TNM staging remains prognostic – informing treatment selection but not predicting response.

Technological advances in molecular biology and histopathology techniques, supporting well-designed studies, have greatly increased our understanding of the molecular basis of tumor biology, progression, and treatment response. Notably since the year 2000, there have been over 80,000 publications in PubMed with the joint headings of ‘cancer’ and ‘predictive marker’, indicating the growing role of predictive tools. However, patient benefit has not fully materialised – the list of predictive tools routinely used in the clinical setting is still very limited, in contrast to numerous prognostic tools. At the end of 2019, 64 antitumor therapies targeting 24 molecular alterations were in clinical practice; detection of the alteration was required for prescription of only 19 (30%) of these therapies [5].

The identification and development of most currently available biomarkers utilised tumor biopsy specimens and this remains the primary method of tumor molecular assessment in routine clinical practice. However, the panacea of a non-invasive blood test – a ‘liquid biopsy’ – that can provide tumor-specific multi-omic information has garnered considerable interest and investment over the past few decades. Primary areas of focus in liquid biopsy research have been circulating tumor DNA (ctDNA) and circulating tumor cell (CTC) compartments, which are discussed below.

In this review we discuss the current status of predictive tools for conventional and targeted therapy, highlighting barriers to their development and widespread application. We also outline outstanding questions and unmet needs regarding future directions of predictive biomarker development and clinical adoption.

## 2 What predictive tools are currently in clinical practice?

**Fig. 1:**
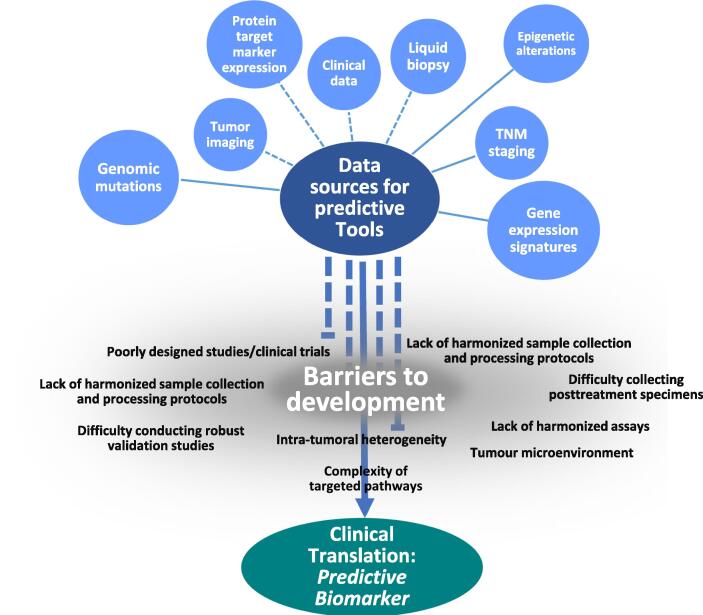
Pictorial representation of data sources for predictive biomarker development and barriers that prevent successful clinical translation. Individual data variables (blue) may be predictive but some may be prognostic (such as TNM) but in combination form a predictive tool.

When discussing ‘conventional therapy’ in this review we are referring to surgery, chemotherapy, or radiotherapy. As will be highlighted, predictive biomarkers are lacking for these treatments. Stepping back, it is not difficult to see why. At the time of their development and introduction, these conventional therapies were the only option for cancer patients and, despite advances in research, have been the mainstay of cancer therapy for over half a century. For this reason, there was no urgent clinical need for predictive biomarker development and so research mainly focused on improving their efficacy whilst decreasing morbidity, such as intensity-modulated radiotherapy [6]. Contrast this to novel targeted or immunotherapy drugs, which have variable inter-patient efficacy and considerable cost implications. For these new therapies predictive biomarkers are important, if not essential, as companion diagnostics (CDx), to enable patient selection and so increase efficacy and cost-efficiency. In this section we will discuss the various types of existing and potential predictive biomarkers for conventional and targeted therapies. We will highlight key examples to provide context for further discussion of barriers to development and clinical translation (see Fig. 1).

### 2.1 Predictive tools guiding surgical intervention

Few studies have sought to define predictive biomarkers related to surgical intervention. The majority of evidence remains prognostic in nature, discussing surgical outcomes irrespective of the treatment decision. In cancer types where surgery is established as the primary treatment modality, trial design to define predictive biomarkers for patients who should or should not undergo surgery is ethically challenging [7]. In those cancers with equivocal evidence for primary treatment then predictive biomarkers are urgently needed. As discussed below, research has largely focused on the adjuvant setting. However, advances in surgical technique and technology promise improved outcomes – this is particularly true in cancer types where surgical access is difficult and creates significant patient morbidity. In this setting, arguably robotic surgery has the greatest potential for impact, for example surgical robotic endoscopic surgery [8] or trans-oral robotic surgery to treat oropharyngeal cancer [9]. However, such technical advances are often not developed with accompanying predictive biomarkers and cohort sizes in early phase trials are small.

One area that has garnered interest is that of enhanced or modified tumor imaging to guide surgical decision making in a predictive fashion [10]. In the assessment of draining lymph node basins from primary tumors, functional imaging modalities that measure tissue physiology can predict the need for surgical intervention, such as axillary or cervical node clearance in breast cancer (BRC) and head and neck squamous cell carcinoma (HNSCC) respectively [11], [12]. For example, Mehanna et al demonstrated the predictive value of positron emission tomography-computed tomography surveillance in HNSCC patients post-chemoradiotherapy, with surgical intervention limited to those who had residual disease [13].

### 2.2 Predictive biomarkers for conventional radio- and chemotherapy

Predictive biomarkers to identify patients who will benefit from adjuvant radio- and/or chemotherapy are crucial to improve outcomes, especially in the primary surgery setting. As will be discussed, several tools have been investigated to develop predictive biomarkers for this purpose – including biomarkers related to mechanism of action, gene expression signatures, residual disease and liquid biopsies. We give key examples of each to highlight the current landscape.

Biomarkers of the mechanism of action of radio- and/or chemotherapy are an obvious candidate for predictive utility; identifying protein or gene expression in downstream pathways directly related to the treatment modality. One such example, is excision repair cross completing group 1 (ERCC1) protein, a component of the DNA repair pathway, which has been investigated as a biomarker of chemotherapy response [14], [15], [16]. While ERCC1 demonstrated prognostic value for multiple cancers, clear evidence for its utility as a predictive biomarker is lacking. For example, early promise as a predictive biomarker for adjuvant chemotherapy in NSCLC [15] or chemotherapy efficacy in CRC [17] was not reproduced in larger cohort studies. Such evidence demonstrates the challenge to identify and translate predictive biomarkers to clinical practice.

A recent systematic review identified 10 potential predictive biomarkers of radiotherapy response [18]. Of these, five were protein markers of DNA damage response and five were gene signatures. The closest biomarker to clinical translation was the radiosensitivity index (RSI), comprising 10 genes whose expression significantly correlated with tumor cell radiosensitivity [19]. The RSI has been clinically validated in multiple patient cohorts including different cancers, the largest being breast cancer (n = 503) [20]. Given the interdependency of radiosensitivity and oxygen availability, gene signatures for assessment of tumor hypoxia have been developed for multiple cancers, including HNSCC [21], [22]. Retrospective analyses support the utility of such signatures to predict benefit from hypoxia modification, further validation is ongoing [23].

Combining expression profiles from multiple genes into validated panels has facilitated the development of numerous predictive tools. One of the earliest and most widely adopted examples is Oncotype DX – a 21 gene signature initially developed to predict recurrence in node-negative tamoxifen-treated breast cancer (BRC) [24]. Subsequently, Oncotype DX has been shown to predict benefit from chemotherapy in high-risk, but not low-risk patients. A recent trial of 9,719 HER2-negative node-negative BRC patients demonstrated that endocrine treatment was non-inferior to chemotherapy plus endocrine treatment for patients with a mid-range Oncotype DX score of 11–25 (n = 6,711), thus predicting those patients who can be spared adjuvant chemotherapy [25]. The MammaPrint assay has further developed this concept into a 70 gene signature that predicts recurrence in node-negative BRC patients irrespective of estrogen receptor or HER2 status [26]. In contrast to Oncotype DX, which uses RT-PCR to quantify gene expression, Mammaprint uses a microarray assay, which can assess expression of thousands of genes, allowing a depth of information previously unobtainable within clinical cost and time constraints.

The above-described predictive tests are tissue-based, often utilising formalin-fixed paraffin-embedded (FFPE) tumor samples for the quantitative assessment of transcript abundance or protein expression. Alternative strategies include functional/molecular imaging to predict treatment response and liquid biopsy-based assessments. The presence of residual disease following primary treatment can be regarded as a predictive biomarker for specific adjuvant therapy. Using BrC as an example, the CREATE-X trial demonstrated that HER2-negative residual disease following neo-adjuvant chemotherapy and primary surgery was a marker of response to adjuvant capecitabine [27]. Similar results were observed in the KATHERINE trial, whereby HER2-postive residual disease was a predictive biomarker for response to trastuzumab emtansine (T-DM1) adjuvant therapy [28]. The utility of liquid biopsies (primarily blood samples) is being widely explored – mostly in relation to targeted therapies (discussed below) and immunotherapies [29], [30]. Very few trials have evaluated ctDNA derived predictive biomarkers for conventional therapies, the main focus being prognostic markers. The COBRA trial is evaluating ctDNA as a predictive biomarker for adjuvant chemotherapy in CRC, but results are still awaited [31].

### 2.3 Predictive biomarkers guiding application of targeted therapies

The advent of targeted therapies, such as tyrosine kinase inhibitors (TKIs), has resulted in numerous predictive biomarkers being developed, based upon expression of the specific therapeutic target. However, there is a disparity between target availability and therapeutic efficacy in certain cancer types. Thus, TKIs have shown success and are a mainstay of treatment in, for example, NSCLC, BRC and CRC. In contrast, EGFR-inhibitors such as cetuximab have not demonstrated clear benefit in HNSCC in the primary setting when compared to standard-of-care platinum-based CRT [32], [33] and are currently approved as an adjunct in recurrent/metastatic patients only [34]. Variability in response within tumor types may be explained mechanistically. For example, KRAS mutations which lead to constitutive activation of downstream signalling pathways, rendering upstream inhibition of EGFR futile. Testing of additional predictive biomarkers is required to identify such contraindications, for example KRAS genotyping in metastatic CRC patients to predict response to Cetuximab therapy [35].

The Food and Drug Administration (US) (FDA) currently lists 44 CDx devices/tools approved in oncology [36]. Tests have been approved for single gene mutations serving as predictive biomarkers in multiple companion diagnostic applications, for example BRACA1/2, ALK, EGFR, KRAS and BRAF mutations. The development of next-generation sequencing and high-throughput assays assessing multiple gene expression or mutation patterns has led to a plethora of expression signatures and mutation panels reported to have predictive value. Several of these have now been translated into routine clinical practice. One example is the FDA approved FoundationOne CDx, a tissue-based test which analyses mutations in 324 genes, in addition to providing microsatellite instability (MSI) and tumor mutational burden (TMB) scores [36]. Approved as a CDx for over 20 targeted therapies, FoundationOne CDx demonstrates how improved cost-effectiveness of next-generation sequencing has transformed genomic testing for predictive biomarkers in clinical practice. More recently, this test has been developed for analysis of ctDNA from blood samples – the FoundationOne Liquid CDx [37] – which directs the use of targeted therapies in NSCLC, prostate, breast and ovarian cancer. Several ‘single gene’ ctDNA tests are also approved, such as the cobas EGFR Mutation Test for the detection of EGFR mutations in NSCLC to predict sensitivity to Osimertinib [38], [39]. However, their clinical application is mostly limited to trials, whereas the FFPE tissue-based counterparts are more commonly used.

### 2.4 Predictive tools of toxicity

Pharmacogenomics is the study of how genes affect an individual’s response to drugs. It combines pharmacology and genomics to develop safe effective medications, tailoring dosage to a patient’s genetic profile. This is particularly important because combination approaches based on tumor biology – for example blockade of multiple aberrant signalling pathways – may result in enhanced toxicity which precludes their use [40]. In the context of this article, we highlight the application of pharmacogenomics as a predictive tool for the safety of oncological treatment. For example, dihydropyrimidine dehydrogenase (DPYD) genotyping is approved for prediction of fluorouracil (5-FU), capecitabine or tegafur treatment toxicity [41], [42]. However, testing is not widely adopted in clinical practice. Further markers include thiopurine S-methyltransferase (TPMT) and catechol O-methyltransferase (COMT) variants associated with cisplatin-related hearing damage in frontline paediatric cancer treatment [43], [44], [45]. Table 1 summarises the key predictive markers that should be assessed prior to prescription of specific oncology treatments to minimise associated toxicities. Revised labels are updated often by regulatory bodies to include approved tests for markers of toxicity, but there is always some delay in clinical adoption.

**Table 1:** FDA-Approved oncology drugs with labels that have been revised to include Toxicity predictive markers [43], [44], [46].

Drug	Year of treatments’ FDA Approval	Predictive Biomarker
Capecitabine	1998	DPYD
Cisplatin	1978	TPMT poor metabolisers
Fluorouracil	2000	DPYD
Irinotecan	1996	UGT1A1
Mercaptopurine	1953	TPMT poor metabolisers
Nilotinib	2007	UGT1A1
Pazopanib	2009	UGT1A1
Rasburicase	2002	G6PD
Sebrafenib	2018	G6PB
Tamoxifen	1977	CYP2D6 poor metabolisers
Tamoxifen	1977	F5; Factor V Leiden carriers
Tamoxifen	1977	F2; Prothrombin mutation G20210A
Thioguanine	1966	TPMT poor metabolisers

CYP2D6, Cytochrome P450 2D6; DPYD, dihydropyrimidine dehydrogenase; G6PD, glucose-6-phosphate dehydrogenase; F2, coagulation factor II; F5, coagulation factor V; TPMT, thiopurine S-methyltransferase; UGT1A1, UDP glucuronosyltransferase 1 family, polypeptide A1.

## 3 Translational research and clinical adoption

Notwithstanding the successful applications described above, few predictive biomarkers have fulfilled their promise to date – translating from discovery to clinical utility [47], [48]. In this section we discuss the various underlying issues that hinder the development and wider clinical adoption of effective predictive tools. We highlight the shortfalls of poorly designed and underpowered studies, the innate difficulty in undertaking sufficiently large robust validation studies, as well as the need for universally harmonised sample collection protocols and assays.

### 3.1 Study design, sample size and statistics

The concepts of alternative hypothesis testing and statistical power were first formalised by Neyman and Pearson in 1928 [49]. Almost one hundred years later, lack of statistical power remains a common confounding factor for the interpretation of study results. Sample size calculations depend on the selection of a single endpoint. However, in practice, clinical studies often have multiple endpoints, and indeed any sample size can be justified by prudent choice of endpoint and power. Issues arise when investigators do not determine a meaningful effect size prior to study initiation [50], [51], [52], [53], [54]. A power calculation forces investigators to name the main outcome variable of their trial, which can then be checked in the analysis, to protect against data dredging [50]. Underpowered studies can create major barriers to biomarker validation and downstream clinical adoption.

Early phase biomarker studies sometimes lack epidemiological validity or statistical power and therefore fail to detect a difference between groups even where such difference exists. Paradoxically, insufficient statistical power also increases false positives, as well as false negatives [51], [52]. A recent study [55], reported discrepancies between primary outcomes in published articles versus original study protocols for 62% of trials reviewed. Hence, publication bias favours reporting of statistically significant results. The combination of underpowered early studies and reporting bias can negatively impact publication of large validation studies, especially if results are non-significant [48], [51]. Thus, appropriate early trial design, with well-planned and executed recruitment strategies are paramount for robust, successful biomarker studies. The development and validation pathway should be designed to meet the specific performance criteria for different biomarker applications, such as treatment selection versus disease monitoring.

Another common pitfall in study interpretation is the application of multiple statistical analyses to the same data sets, hence increasing the chance of false positives [53]. By multiple testing, we refer to instances when a dataset is subjected to repeat statistical testing – including multiple time-points or subgroups – all of which increase the probability of detecting a false-positive. Meta-analyses and good accompanying clinical data can help strengthen studies. However, confounding factors such as diverse treatment options/delivery schedules or individual patient characteristics, can make it more difficult to avoid statistical errors and fully control the planning of analyses. To prevent these serious problems, planned comparisons should be pre-specified in the research protocol, with adjustments for multiple testing.

Retrospective studies are frequently used for early-stage biomarker development and validation being time and cost-effective. However, they are often subject to bias, such as control selection, outcome selection, loss to follow-up and differential diagnosis. Another weakness is the difficulty in ascertaining whether the analyses used were designed when the research idea was conceived or were a result of data dredging and p value hunting for hypothesis redevelopment. Good practice for navigating through some of the common pitfalls can be found in the following reviews [56], [57].

### 3.2 Lack of standardised, harmonised sample collection and processing protocols

Historically, biomarker discovery and development has lacked the well-defined regulatory structure mandated for the development of new drug entities. The resultant lack of pre-analytical studies, harmonised sample collection and standardised assay protocols across clinical laboratories, contributes to diminished reproducibility of study findings and undermines downstream clinical application. For example, poor-quality clinical samples (due to collection or storage practices and sample age) can contribute to false discovery, even when using a meticulous study design [58]. These problems render many markers insufficiently sensitive or specific for their intended use in clinical practice. Pepe and colleagues proposed a five-phase formal categorization to guide biomarker development [59]. Subsequently, the establishment of the Early Detection Research Network (EDRN) by the National Cancer Institute, USA., has led to improved coordination between biomarker research laboratories [60]. The application of uniform standards should facilitate the translation of newly discovered biomarkers to the clinic [59], but this process must be enforced by all approval bodies for global compliance and standardisation [60].

In addition to sample collection issues, the lack of standardised, robust assays often creates issues in meta-analysing studies and interpreting results, as well as identifying reliable prediction tools. Taking an example from immunotherapy approaches, multiple tests are approved for assessing expression of the immune checkpoint, programmed death ligand-1 (PD-L1). The assays use different platforms, antibodies, scoring systems and cut-offs for positivity [61], different tests being aligned to specific indications. Even though all are assaying the same marker (PD-L1) they are effectively separate tests with limited transferability [62], [63]. Even where a single biomarker test is employed, inter-laboratory variation may confound assay standardisation. One way to address this issue is via centralised testing, where only a single laboratory offers a specific test (for example, the FoundationOne CDx and FoundationOne liquid CDx).

As alluded to above, issues arise when quantification is a requirement for the predictive tool and cut-off values need to be implemented [64]; both the lowest and highest levels of quantification must be determined for sensitivity and specificity, to enable clinical adoption within the given application context. Most times these are not harmonised between different studies, making interpretation challenging and leading to poor clinical adoption. One root cause for this challenge arises from the use, in early development, of a cohort that is defined by a certain genetic or environmental background (in order to have sufficient disease homogeny to develop and assess a molecular biomarker) thus introducing a sample bias [48], [50], [53]. Such bias becomes an issue when validating predictive tools developed in a well-defined study population and compounded when translating such biomarkers into clinical practice – where every individual patient poses unique challenges to the biomarker: tumor location and heterogeneity, co-morbidities, lifestyle and environmental influences, as well as an individual clinical care team with local limitations or care choices [54], [65].

### 3.3 Difficulty in undertaking validation studies of sufficient size and robust design

Trial recruitment is always an issue when conducting biomarker validation studies, even in cancer types with a higher population incidence. This problem is accentuated for less common cancers, like HNSCC, and is further compounded when assessing biomarkers of low incidence. Large scale, multi-centre studies are needed to address this. One such example is the phase II NCI-MATCH trial which recruited a large proportion of patients with less common (e.g. gastroesophageal, kidney), as well as the most frequent cancers (e.g. colorectal, breast, NSCLC) from over 1100 sites across the United States [66].

Another approach is the so-called adaptive trial design, which has no formal pre-trial sample size calculation [67]. This is essentially a two-phase trial, with an initial phase used to identify optimal drug dose, biomarker cut-offs, or estimates of the standard deviations of the outcome variables. Such data can then inform appropriate changes to the trial protocol, including amended statistical power calculations and target sample size. This approach is gaining significant traction in cancer research, an example of which is the UK Lung Matrix trial. Patients are stratified into different treatment arms according to genotype markers that have been identified in their cancer. Of 5,467 patients screened, 2,007 were eligible for enrolment and of these only 288 patients received genotype-matched therapy [68]. However, in clinical practice this would facilitate targeted treatment of patients who would benefit from the intervention, minimising potential side-effects and costs.

### 3.4 Poor clinical uptake of available predictive tools

As discussed above, effective predictive tools are available at least for some indications. However, availability does not necessarily correlate with clinical adoption. For example, national guidelines recommend RAS, BRAF and MSI-testing for patients with metastatic colon cancer in the United States, but the testing rate is only around 50% [69]. A similar situation has been reported for NSCLC, where testing rates, although improving, still vary for individual markers and between countries [70]. Barriers to testing include limited sample availability, complexities of test selection, timelines (where treatment is urgently required and particularly if single marker tests are sequentially applied), cost, and difficulties in interpreting and applying test results. Targeted NGS approaches (such as FoundationOne CDx) provide broad information covering multiple actionable targets and are cost-effective. The downside is increased complexity of data generated and the associated challenges in clinical application. The European Society for Medical Oncology (ESMO) has developed a framework to facilitate prioritisation of genomic targets based on clinical evidence of utility – the ESMO Scale for Clinical Actionability of molecular Targets (ESCAT) [71]. However, there remains an unmet need for a comprehensive support platform to uniformly match NGS results with therapies for cancer patients [72].

## 4 Cellular and molecular tumor heterogeneity

In the following sections we consider tumor-intrinsic and microenvironmental effects that impact the development and/or use of predictive tools, along with ongoing research to overcome such hurdles.

### 4.1 Intratumoral heterogeneity and difficulties of on- or post-treatment sample collection

As previously noted, most predictive tools – including 32 out of 37 FDA-approved CDx for solid tumors [36] – are exclusively tissue-based. The invasive nature of such tests precludes multiregional or serial sampling for most indications, meaning that treatment decisions are based on a single diagnostic sample and could introduce a sampling bias. Many tests also involve bulk, rather than single cell analyses, and therefore do not assess whether all or only a proportion of cells (usually tumor cells) are positive for the marker of interest. Consequently, intratumoral heterogeneity (ITH) can be a major confounding factor. ITH was originally defined as the uneven spatial or temporal distribution of genomic alterations within an individual tumor. This has expanded to include epigenetic, transcriptomic and proteomic diversity within tumor cells, as well as their interaction with the microenvironment (TME) and diversity of the TME itself (discussed below).

From a gene-centric viewpoint, tests based on a single tissue sample may only capture a snapshot of the genomic diversity present within the whole tumor [73]. Using multi-region sequencing of clear cell carcinomas, Gerlinger et al. showed that many driver mutations are subclonal and ITH increases with the number of biopsies analysed [74]. Targeted treatments may select for tumor cells lacking the specific genomic alteration, or those containing compensatory changes, leading to treatment resistance [75]. The limitations of single sample-testing and ITH may be further compounded by frequent use of single marker testing. In recent years there has been a gradual progression from single marker to multi-locus testing – paralleling the development of NGS technology. This enables ‘one-step’ selection of the most appropriate single target. It also facilitates identification of combination approaches targeting multiple pathways with decreased capacity for acquired resistance.

Liquid biopsies may help to address both spatial and temporal ITH. With respect to the latter, such minimally invasive techniques are well-suited to serial sampling, enabling on-treatment monitoring and post-treatment assessment. Multiple studies have evidenced the utility of ctDNA to track the temporal heterogeneity of resistance mechanisms and acquired mutations in advanced breast, ovarian, lung and gastrointestinal cancers [76], [77], [78]. However, whilst liquid biopsies have shown high specificity, their sensitivity may be lower than that of tissue-based approaches [79], [80]. It is noteworthy that tissue-based testing is recommended if all test results for the FoundationOne Liquid CDx are negative [37].

The relative merits of ctDNA versus CTCs to address ITH are not fully resolved. Several papers have highlighted ctDNA as a more accurate assessment of disease burden or tumor mutational profile when compared to CTCs [81]. Like bulk tissue-based tests, ctDNA analysis does not address the proportion of tumor cells containing specific alterations. Assessment of CTCs, whilst more technically challenging, enables evaluation of genomic variation at the single cell level. For example, single-CTC RNA-sequencing from prostate cancer patients has identified androgen receptor gene mutations correlated to disease progression [82], predicting patients who would fail androgen inhibitor treatment. Improvements in CTC sequencing and multi-parameter characterisation hold promise for predictive biomarker development. The CellSearch platform remains the only FDA approved CTC enrichment device; however, the reliance upon single marker (EPCAM)-positive cell selection has intrinsic bias which may limit clinical utility [83]. Cell size/deformability-based technologies, such as microfluidic enrichment, seek to address this but have their own limitations, such as lower sample purity [84]. Representing the latter approach, the Parsortix (Angle Plc) microfluidic CTC enrichment device is currently under FDA review for use with metastatic BRC patients.

### 4.2 Tissue microenvironment effects

As noted above, ITH exists at multiple levels, including within TME components. TME interactions have important roles in tumor cell survival, proliferation, differentiation, and metastasis. Effects can be mediated via direct cell-cell contact or the plethora of cytokines, chemokines and growth factors produced by diverse cell types within the TME – including pro-tumoral cancer-associated fibroblasts or suppressive immune subsets (myeloid derived suppressor cells, tumor associated macrophages, regulatory T cells, etc.) and anti-tumoral immune effector cells (T cells, NK cells, type I macrophages, etc.). Other variable features of the TME that impact on treatment response include nutrient and oxygen availability. As discussed above, hypoxia negatively impacts radiotherapy response; beyond this, it selectively disadvantages anti-tumoral immune cells within the TME [85]. Poor vascularisation – an important contributory factor to tumor hypoxia – also limits entry of both immune cells and chemotherapy agents. Although tumor-TME interactions are critical determinants of treatment response and outcome, they are given minimal consideration by current predictive tools, which often focus on intrinsic properties of tumor cells. Only for immunotherapy (discussed elsewhere in this issue) is due consideration given to the role of the TME. In vitro model systems – the focus of this special issue – provide the best opportunity to explore the complexity of tumor-TME interactions and their effects on treatment response.

### 4.3 Complexity of pathways or processes that are being therapeutically targeted

Poor response to a targeted agent despite therapeutic biomarker matching is often a reality, as highlighted with EGFR inhibition in HNSCC [86]. A further example can be drawn from immunotherapy approaches, where a recent review concluded that across 15 tumor types, tumor PD-L1 expression was predictive of response to immune checkpoint blockade in less than 30% of cases [63]. Conversely, some patients derive benefit from PD-1/PD-L1 blockade in the absence of PD-L1 expression [87], thus highlighting the need for more robust predictive biomarkers in certain treatment groups.

The underlying mechanism behind such treatment resistance may be attributed to several confounding factors including: partial or incomplete pathway inhibition, biochemical plasticity in response to drugs, the presence of co-occurring driver mutations or spatial heterogeneity of tumor cells lacking the targeted marker [88]. Furthermore, the structure and function of the treatment target proteins are regulated by multiple molecular factors, such as posttranslational modification, which are often not assessed with conventional tests i.e. phosphorylated proteins in their activated form. Compensatory pathways may explain why certain treatments fail in some cancer types [89]. For example, resistance to EGFR-inhibitors due to compensatory MAPK, PI3K/ATK and STAT pathway activation [90] or co-occurring alterations in CTNNB1 and PIK3CA in lung cancer [91]. The ongoing discovery of such alternative pathways serves to highlight our limited understanding of complex cellular oncogenic mechanisms. In the NCI-MATCH trial ~38% of patients with actionable alterations were excluded from treatment due to co-occurring resistance mutations [66].

In many solid tumors, mutations in oncogenes – such as the RAS family – are the key drivers of survival, proliferation, etc. However, this is not universal. For example, in HPV-negative HNSCC key genetic changes involve loss of tumor suppressor function (TP53, CDKN2A), rather than activation of oncogenes [92]; such changes may be prognostic but are not actionable. In such situations, the lack of a therapeutic target makes predictive biomarker development particularly difficult.

## 5 Outstanding questions and future direction

Predictive biomarkers can greatly improve treatment selection and ultimately patient outcomes, as well as ameliorate side-effect profiles in cancer therapy. The rapid evolution of clinically adopted tests and molecular biomarkers, in particular NGS, and better understanding of cancer biology and disease progression will allow clinicians to provide treatments that are patient stratified, precise and better tolerated. Oncology practice will increasingly be ruled by cost effectiveness in clinical management and this is an area where effective predictive tools can come into their own. For this to materialise predictive tools need to be easy-to-apply, relatively inexpensive, robust, and reliable. Furthermore, clinicians need to be able to interpret the outcomes and to have treatment selection options that are appropriate and approved. Moreover, advances in technology would allow for rapid ‘table-top‘ evaluation of at least some predictive biomarkers to take place during consultations, speeding up disease assessment and treatment enrolment. Table 2 highlights outstanding questions that must be considered when future research seeks to develop successful predictive tools for clinical translation.

**Table 2:** Outstanding questions and research/clinical needs still to be addressed for successful development of biomarkers and implementation of predictive tools into clinical practice.

Predictive tools the Outstanding questions/needs
• Can effective predictive tools be developed using clinical data/factors that are routinely recorded/measured, e.g. age, gender, T/N/M, blood counts, blood proteins, scans, BMI, co-morbidities, etc.? – as no/less requirement for high-level technologies and minimal add-on costs, may be more universally applicable.
• Can we establish and support large-scale collaborative projects – especially for rare cancers or subtypes – to generate large, robust datasets for validation of predictive tools and use of AI-based machine learning for analysis, hence produce simplified outputs to facilitate clinical implementation?
• Are biomarkers and development models population biased, and can biomarkers be universally applied between genetically diverse populations?
• Can licensing agencies demand and enforce the use of companion biomarkers that direct treatment?
• Large datasets and multiple layers of clinical data, in particular NGS, for biomarker discovery and patient clinical assessment pose ethical concerns that need to be addressed. How we safeguard patient data and minimise the risk of deanonymizing data sets?
• There is a need for development/application earlier in the treatment timeline. Predictive tools are mostly developed in advanced disease settings – is this problematic for wider adoption?

The aforementioned issues of genomic and transcriptomic ITH, the TME and compensatory pathway activation are all intertwined, contributing to a multifactorial picture of resistance to conventional and targeted therapy. No single predictive biomarker is likely to have the appropriate power to direct treatment decisions with clinical benefit in cancers with such great heterogeneity. Therefore, the future of cancer predictive tools may require an amalgamation of several diverse markers that can give a more actionable molecular staging that will indicate the outcome of treatment response. As evidence for the utility of liquid biopsies continues to grow, the evaluation of ctDNA and CTCs will be a key area of future research to contribute to predictive tools.

Finally, as research continues to address the obstacles of design and delivery of predictive biomarker trials, the greatest challenge will be the analysis of vast ‘omics’ datasets derived from high dimensional tumor characterisation and NGS. To this end, integrative multi-omics and the development of AI algorithms to mine vast quantities of data is undoubtedly the future of predictive biomarker development.

## Declaration of Competing Interest

The authors declare that they have no known competing financial interests or personal relationships that could have appeared to influence the work reported in this paper.
